# Distance-based evaluation of tumor budding in colorectal cancer

**DOI:** 10.1007/s00428-026-04471-9

**Published:** 2026-03-06

**Authors:** Ville K. Äijälä, Päivi Sirniö, Henna Karjalainen, Meeri Kastinen, Vilja V. Tapiainen, Hanna Elomaa, Maarit Ahtiainen, Vesa-Matti Pohjanen, Taneli T. Mattila, Outi Lindgren, Olli Helminen, Erkki-Ville Wirta, Jukka Rintala, Sanna Meriläinen, Juha Saarnio, Tero Rautio, Toni T. Seppälä, Jan Böhm, Jukka-Pekka Mecklin, Anne Tuomisto, Markus J. Mäkinen, Juha P. Väyrynen

**Affiliations:** 1 Translational Medicine Research Unit, Medical Research Center Oulu, Oulu University Hospital, and University of Oulu, Oulu, Finland; 2 Research Program in Systems Oncology, University of Helsinki, Helsinki, Finland; 3 Central Finland Biobank, Hospital Nova of Central Finland, Well Being Services County of Central Finland, Jyväskylä, Finland; 4 Department of Gastroenterology and Alimentary Tract Surgery, Tampere University Hospital, Tampere, Finland; 5 Faculty of Medicine and Health Technology, Tampere University and Tays Cancer Centre, Tampere University Hospital, Tampere, Finland; 6 Department of Gastrointestinal Surgery, Helsinki University Central Hospital, University of Helsinki, Helsinki, Finland; 7 Applied Tumor Genomics, Research Program Unit, University of Helsinki, Helsinki, Finland; 8 Department of Pathology, Hospital Nova of Central Finland, Well Being Services County of Central Finland, Jyväskylä, Finland; 9 Department of Education and Research, Well Being Services County of Central Finland, Jyväskylä, Finland; 10 Faculty of Sport and Health Sciences, University of Jyväskylä, Jyväskylä, Finland

**Keywords:** Tumor budding, Colorectal cancer, Prognosis, Cohort

## Abstract

Tumor budding is an established adverse prognostic factor in colorectal cancer (CRC), based on the number of isolated single tumor cells or small tumor cell clusters at the invasive front. While bud counts are well studied, the prognostic significance of the spatial distribution and distance of tumor buds away from the tumor bulk is unclear. We defined TB-distance as the average distance from the tumor bulk to the three farthest tumor buds and evaluated its clinicopathologic and prognostic associations in two independent CRC cohorts (N = 776 and N = 1,100). Using a cohort-derived cutoff, high TB-distance (≥ 123 µm) was significantly associated with adverse tumor characteristics, including high grade, advanced disease stage, lymphovascular invasion, high conventional tumor budding grade, and MMR proficient status (p < 0.003 for all). High TB-distance was also associated with shorter cancer-specific survival (Cohort 1: multivariable HR (high vs. low) 1.47, 95% CI 1.04–2.09, p = 0.030; Cohort 2: multivariable HR 1.34 95% CI 1.04–1.74, p = 0.026). However, TB-distance did not provide additional prognostic information within conventional tumor budding grade strata or when modeled alongside tumor budding. These findings indicate that high TB-distance is associated with aggressive tumor morphology and worse outcome but does not improve prognostication beyond standard tumor budding assessment. TB-distance may still be useful as a visual aid in routine pathology and a quantifiable spatial feature for computational pathology.

**Supplementary Information:**

The online version contains supplementary material available at https://doi.org/10.1007/s00428-026-04471-9.

## Introduction

Colorectal cancer (CRC) is the second leading cause of cancer-related death worldwide, with more than 1.9 million new cases diagnosed annually and a mortality rate of 9.3% [1]. Five-year survival varies by stage, exceeding 90% in early-stage disease but falling below 10% in metastatic CRC [2, 3]. Accordingly, the treatment regimen can range from endoscopic resection or surgical removal and chemoradiotherapy to advanced approaches such as immune checkpoint inhibitors and targeted therapies [4–6]. However, the traditional Tumor-Node-Metastasis classification does not fully predict which patients will benefit from adjuvant therapy, as some stage II tumors exhibit recurrence risks comparable to or even greater than those of some stage III cancers [7]. This limitation has motivated the use of additional prognostic markers, including lymphovascular and perineural invasion, mismatch repair deficiency, and tumor budding, to refine risk stratification and inform clinical decision making [7–9].

Tumor budding is a prognostic marker defined as single tumor cells or small clusters of up to four cells located at the invasive margin of the cancer [10]. Tumor budding is thought to reflect the initial steps of tumor invasion and metastasis, as cells detach from the main tumor mass at the invasion front [11]. Consistent with this concept, numerous studies have identified tumor budding as a strong indicator of poor prognosis in CRC [12–16]. Clinically, tumor budding assessment helps guide decisions regarding lymphadenectomy during pT1 tumor endoscopic resections and the selection of high-risk stage II CRC patients for adjuvant therapy [7, 17]. According to the International Tumor Budding Consensus Conference (ITBCC) criteria, tumor budding should be evaluated by counting tumor buds within hotspots on hematoxylin & eosin -stained slides [10]. CRCs exhibiting high-grade tumor budding (≥ 10 buds in 0.785 mm^2^ hotspot) are often considered in decisions regarding adjuvant treatment, whereas intermediate-grade tumor budding (5–9 buds) currently does not routinely influence therapeutic decision-making.

**Fig. 1:**
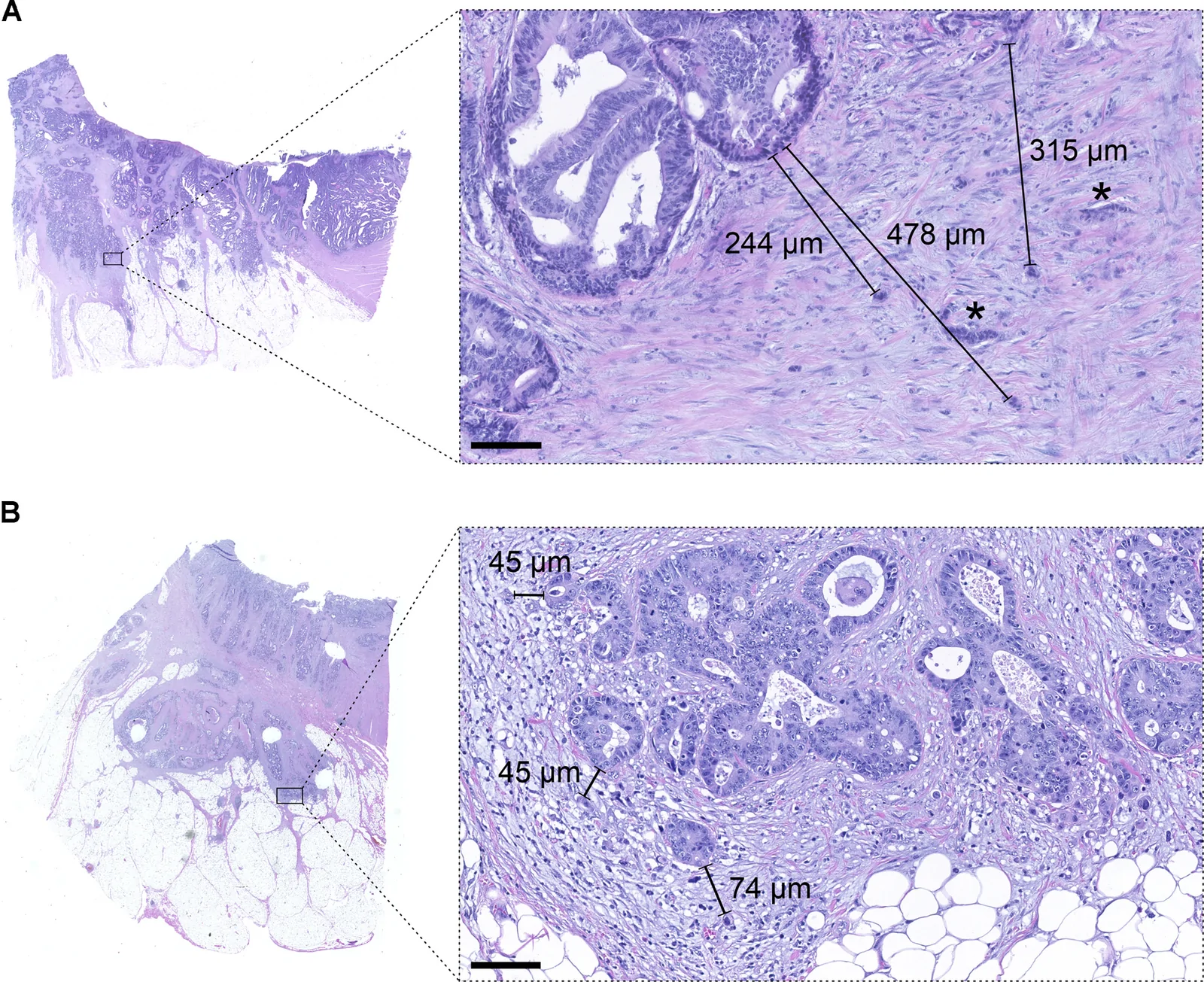
Histological features of high and low TB-distance in colorectal cancer. **A** Hematoxylin and eosin-stained section showing tumor buds that extend a long distance away from the tumor bulk. The mean distance from the tumor bulk to the three farthest buds is 346 µm, classifying the case as high TB-distance. **B** Example of a low TB-distance tumor, with a mean distance of 55 µm. Asterisk (*) denotes tumor fragments (here, poorly differentiated clusters) that are not considered part of the tumor bulk for TB-distance measurements. Scalebar is 100 µm

The primary aim of this study was to determine whether the distance that tumor buds extend from the main tumor mass at the invasive margin can serve as an additional prognostic marker in CRC (Fig. 1). As a secondary objective, we examined whether this parameter could help identify and stratify tumors with low (0–4 buds) or intermediate (5–9 buds) tumor budding that exhibit a poorer prognosis than would be expected based on their conventional tumor budding grade.

## Methods

### Study population

Two independent patient cohorts were analyzed. Cohort 1 comprised 1,011 individuals with stage I–IV colorectal cancer who underwent surgical treatment at Oulu University Hospital (2006–2020). Cohort 2 included 1,343 surgically treated colorectal cancer patients from the Central Hospital of Central Finland (2000–2015). Only cases with representative tumor specimens available in the pathology archives were included.

To avoid confounding effects of preoperative therapy on tumor morphology, patients who had received neoadjuvant treatment were excluded (Cohort 1: N = 235; Cohort 2: N = 243). Furthermore, individuals who died within 30 days post-operation were excluded from the survival analyses (Cohort 1: N = 5; Cohort 2: N = 37). Following these exclusions, 776 patients from Cohort 1 and 1,100 patients from Cohort 2 remained eligible for histopathologic evaluation, while survival analyses included 771 and 1,063 patients.

### Histopathologic analysis

Tumor staging was carried out in accordance with the Union for International Cancer Control (UICC) criteria during routine diagnostics. All additional histopathological features were retrospectively evaluated by investigators with expertise in colorectal cancer histomorphology who were blinded to survival outcomes. Histological grading followed the WHO 2019 classification. Mismatch repair (MMR) status and *BRAF* V600E mutation status were evaluated using immunohistochemistry [18]. Tumor budding was assessed as previously described [15], following the recommendations of the ITBCC [10]. Using hematoxylin and eosin-stained whole-slide digital images, tumor budding was scored in a field of 0.785 mm2 by identifying the hotspot containing the highest tumor bud count and classified as grade 1 (< 5 buds), grade 2 (5–9 buds), or grade 3 (≥ 10 buds). Tumor buds were defined as single tumor cells or clusters of up to four tumor cells. Any cohesive cluster of five or more tumor cells was classified as a poorly differentiated cluster and excluded from the tumor bud count.

To quantify bud extension beyond the tumor bulk, the invasive front was systematically reviewed on whole-slide digital images to identify tumor buds, after which the distances from tumor bulk to the three farthest buds were measured digitally from the edge of each bud to the nearest edge of the tumor bulk. Distances were summed and divided by three to obtain the mean maximum bud extension distance (TB-distance). If fewer than three tumor buds were present, the available distances were summed and still divided by three to reduce the influence of a single unusually distant bud.

Tumor bulk was defined as the main contiguous carcinoma cell mass, distinct from isolated single cells or small clusters at the invasive front (tumor buds or poorly differentiated clusters). Separate large tumor islets showing established tumor architecture (e.g., glands or cribriform structures) were also considered tumor bulk. Mucin pools containing tumor cells were considered part of the tumor bulk. Tumor cells within lymphatic or vascular spaces were excluded from both tumor bulk and tumor buds.

### Statistical analysis

Analyses were performed using IBM SPSS Statistics for Windows (IBM Corp. version 31.0). Findings with two-sided p < 0.05 were considered statistically significant.

Associations between TB-distance (low vs. high) and clinicopathologic variables were examined using crosstabulation, with statistical significance determined by the Chi-squared test. Relationships between TB-distance and both cancer-specific survival (CSS) and overall survival (OS) were assessed using the Kaplan–Meier method and Cox proportional hazards regression. CSS and OS were defined as the time from surgery to cancer-related death or end of follow-up, and the time from surgery to death or end of follow-up, respectively. The median follow-up time among censored cases was 7.0 years (IQR 4.7–10.0) in Cohort 1 and 10.0 years (IQR 7.3–10.0) in Cohort 2. Multivariable Cox models included the following prespecified covariates: age (< 65, 65–75, > 75), sex (male, female), year of operation (Cohort 1: 2006–2010, 2011–2015, 2016–2020; Cohort 2: 2000–2005, 2006–2010, 2011–2015), tumor location (proximal colon, distal colon, rectum), disease stage (I–II, III, IV), WHO grade (low-grade, high-grade), lymphovascular invasion (no, yes), MMR status (proficient, deficient), and *BRAF* mutation status (wild-type, mutant). To reduce degrees of freedom, cases with missing *BRAF* data (7 in Cohort 1; 2 in Cohort 2) were grouped with the majority category (*BRAF* wild-type), given the very small number of missing values. No other variables used in the analyses had missing data.

## Results

### Derivation of TB-distance cutoff

Using ROC analysis and Youden’s index for 10-year CSS in Cohort 1, we determined an optimal threshold for TB-distance of 123 µm. Cases with TB-distance < 123 µm were classified as ‘Low’ and those with TB-distance ≥ 123 µm as ‘High’. This cutoff was then applied unchanged to Cohort 2. The mean distance to the three farthest buds showed comparable discrimination for 10-year CSS as the distance to the single farthest bud (AUC 0.63, 95% CI 0.58–0.68 vs 0.62, 95% CI 0.57–0.67, respectively) (Fig. S1). Reproducibility was assessed in 30 cases by VKÄ and JPV, yielding Spearman’s rho of 0.58 for the continuous measure and Cohen’s kappa of 0.43 for the binary classification, indicating moderate agreement.

### Associations with clinicopathologic features

TB-distance was evaluated in 1,876 CRC cases (Table 1). High TB-distance was present in 209 (27%) tumors in Cohort 1 and 199 (18%) tumors in Cohort 2. In both cohorts, high TB-distance was associated with adverse tumor features, including high WHO grade, advanced disease stage, lymphovascular invasion, high ITBCC tumor budding grade, and MMR proficient status (p < 0.003 for all).

**Table 1:** Baseline patient characteristics and their associations with TB-distance in Cohorts 1 and 2

	Cohort 1				Cohort 2			
		TB-distance				TB-distance		
Characteristic	Total N	Low	High	p value	Total N	Low	High	p value
All cases	776 (100%)	567 (73%)	209 (27%)		1,100 (100%)	901 (82%)	199 (18%)	
Sex				0.57				0.88
Female	364 (47%)	262 (72%)	102 (28%)		557 (51%)	455 (82%)	102 (18%)	
Male	412 (53%)	305 (74%)	107 (26%)		543 (49%)	446 (82%)	97 (18%)	
Age (years)				0.011				0.81
< 65	233 (30%)	158 (68%)	75 (32%)		290 (26%)	236 (81%)	54 (19%)	
65–75	285 (37%)	204 (72%)	81 (28%)		381 (35%)	316 (83%)	65 (17%)	
> 75	258 (33%)	205 (79%)	53 (21%)		429 (39%)	349 (81%)	80 (19%)	
Year of operation				0.25				0.46
2000–2005	-	-	-		342 (31%)	273 (80%)	69 (20%)	
2006–2010	155 (20%)	108 (70%)	47 (30%)		353 (32%)	291 (82%)	62 (18%)	
2011–2015	218 (28%)	168 (77%)	50 (23%)		405 (37%)	337 (83%)	68 (17%)	
2016–2020	403 (52%)	291 (72%)	112 (28%)		-	-	-	
Tumor location				0.56				0.39
Proximal colon	323 (42%)	240 (74%)	83 (26%)		536 (49%)	441 (82%)	95 (18%)	
Distal colon	205 (26%)	152 (74%)	53 (26%)		404 (37%)	335 (83%)	69 (17%)	
Rectum	248 (32%)	175 (71%)	73 (29%)		160 (15%)	125 (78%)	35 (22%)	
WHO grade				0.00061				0.0025
Low-grade	665 (86%)	501 (75%)	164 (25%)		882 (80%)	738 (84%)	144 (16%)	
High-grade	111 (14%)	66 (59%)	45 (41%)		218 (20%)	163 (75%)	55 (25%)	
UICC disease stage				< 0.0001				< 0.0001
I	187 (24%)	164 (88%)	23 (12%)		184 (17%)	168 (91%)	16 (8.7%)	
II	253 (33%)	206 (81%)	47 (19%)		408 (37%)	353 (87%)	55 (13%)	
III	251 (32%)	150 (60%)	101 (40%)		355 (32%)	273 (77%)	82 (23%)	
IV	85 (11%)	47 (55%)	38 (45%)		153 (14%)	107 (70%)	46 (30%)	
pT				< 0.0001				< 0.0001
pT1-pT2	229 (30%)	195 (85%)	34 (15%)		225 (20%)	205 (91%)	20 (8.9%)	
pT3-pT4	547 (70%)	372 (68%)	175 (32%)		875 (80%)	696 (80%)	179 (20%)	
pN				< 0.0001				< 0.0001
pN0	455 (59%)	381 (84%)	74 (16%)		626 (57%)	546 (87%)	80 (13%)	
pN1-pN2	321 (41%)	186 (58%)	135 (42%)		474 (43%)	355 (75%)	119 (25%)	
M				0.0001				< 0.0001
M0	691 (89%)	520 (75%)	171 (25%)		947 (86%)	794 (84%)	153 (16%)	
M1	85 (11%)	47 (55%)	38 (45%)		153 (14%)	107 (70%)	46 (30%)	
Lymphovascular invasion				< 0.0001				< 0.0001
No	429 (55%)	361 (84%)	68 (16%)		858 (78%)	742 (86%)	116 (14%)	
Yes	347 (45%)	206 (59%)	141 (41%)		242 (22%)	159 (66%)	83 (34%)	
Tumor budding (ITBCC)				< 0.0001				< 0.0001
0–4	541 (70%)	503 (93%)	38 (7.0%)		827 (75%)	796 (96%)	31 (3.7%)	
5–9	129 (17%)	53 (41%)	76 (49%)		156 (14%)	81 (52%)	75 (48%)	
≥ 10	106 (14%)	11 (10%)	95 (90%)		117 (11%)	24 (21%)	93 (79%)	
MMR status				< 0.0001				0.00069
Proficient	652 (84%)	453 (69%)	199 (31%)		931 (85%)	747 (80%)	184 (20%)	
Deficient	124 (16%)	114 (92%)	10 (8.1%)		169 (15%)	154 (91%)	15 (8.9%)	
*BRAF* status^A^				0.20				0.92
Wild-type	662 (86%)	477 (72%)	185 (28%)		916 (83%)	749 (82%)	167 (18%)	
Mutant	107 (14%)	84 (79%)	23 (21%)		182 (17%)	150 (82%)	32 (18%)	

Abbreviations: UICC, Union for International Cancer Control; MMR, Mismatch repair; ITBCC, International Tumor Budding Consensus Conference

^A^BRAF data missing for 7 patients in Cohort 1 and 2 patients in Cohort 2

### High TB-distance is associated with poor survival

**Fig. 2:**
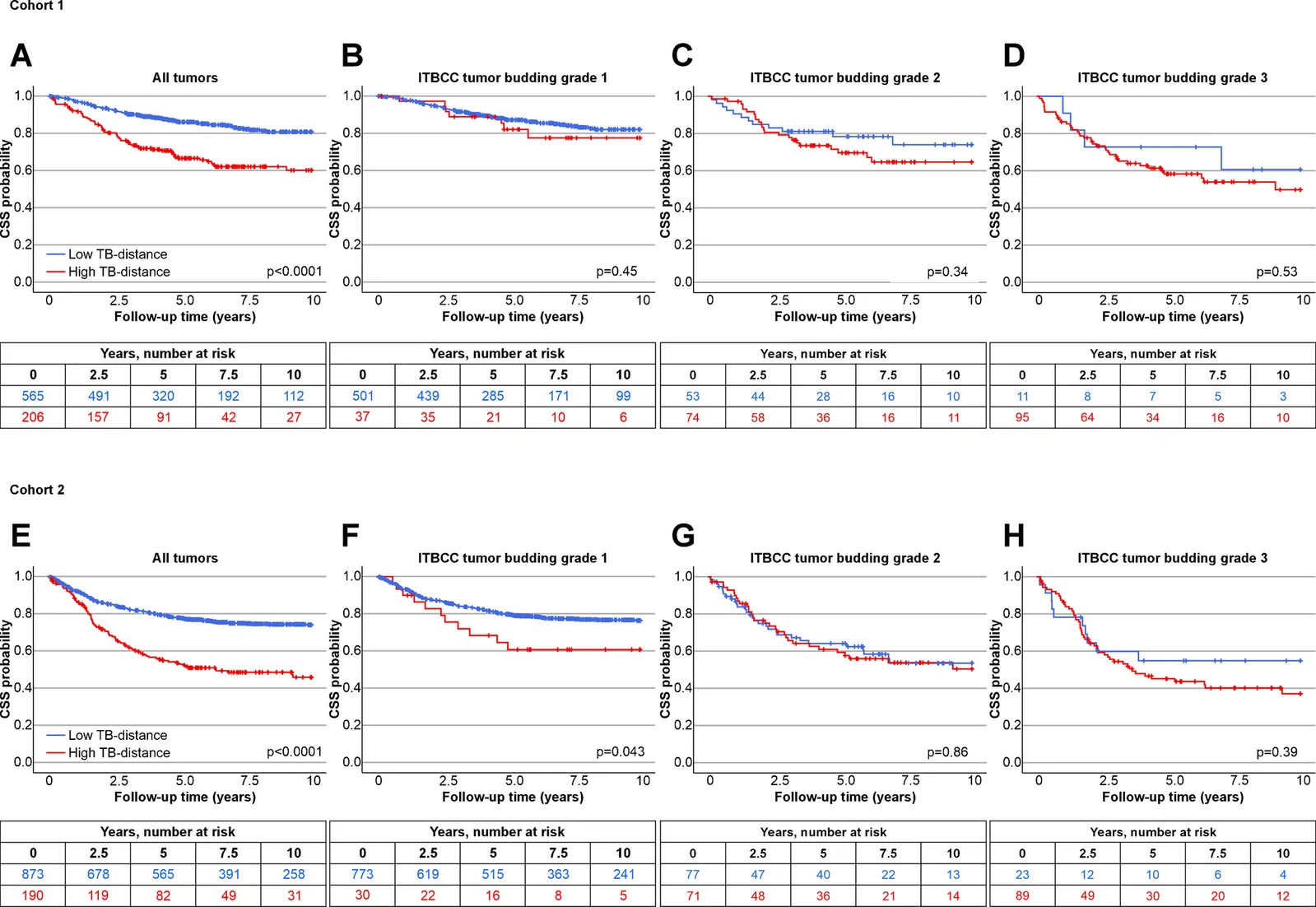
Kaplan–Meier analyses of cancer-specific survival by TB-distance in Cohorts 1 and 2, stratified by ITBCC tumor budding grade. **A**-**D** The association of TB-distance with cancer-specific survival in all tumors (A), tumor budding grade 1 tumors (0–4 buds) (B), tumor budding grade 2 tumors (5–9 buds) (C), and tumor budding grade 3 tumors (≥ 10 buds) (D) in Cohort 1. **E**–**H** The association of TB-distance with cancer-specific survival in all tumors (E), tumor budding grade 1 tumors (0–4 buds) (F), tumor budding grade 2 tumors (5–9 buds) (G), and tumor budding grade 3 tumors (≥ 10 buds) (H) in Cohort 2

In univariable analysis, high TB-distance was associated with shorter CSS and OS in both cohorts (Fig. 2, Table 2). In multivariable Cox models, TB-distance remained independently associated with shorter CSS after adjustment for prespecified clinicopathologic covariates (Cohort 1: HR for high TB-distance 1.47, 95% CI 1.04–2.09, p = 0.030; Cohort 2: HR for high TB-distance 1.34, 95% CI 1.04–1.74, p = 0.026) (Table 2, Table S1).

**Table 2:** Cox proportional hazards regression models for colorectal cancer-specific survival and overall survival according to TB-distance, overall and within ITBCC tumor budding grade subgroups

		Cancer-specific survival			Overall survival		
Variable	No. of cases	No. of events	Univariable HR (95% CI)	Multivariable HR (95% CI)	No. of events	Univariable HR (95% CI)	Multivariable HR (95% CI)
Cohort 1							
TB-distance (all tumors)	771	155			284		
Low	565	85	1 (referent)	1 (referent)	191	1 (referent)	1 (referent)
High	206	70	2.58 (1.89–3.53)	1.47 (1.04–2.09)	93	1.55 (1.22–1.97)	1.37 (1.04–1.81)
p			< 0.0001	0.030		0.00030	0.027
TB-distance (TB grade 1)	538	76			182		
Low	501	69	1 (referent)	1 (referent)	168	1 (referent)	1 (referent)
High	37	7	1.35 (0.62–2.93)	0.99 (0.41–2.39)	14	1.13 (0.65–1.94)	0.97 (0.54–1.74)
p			0.45	0.98		0.67	0.92
TB-distance (TB grade 2)	127	35			47		
Low	53	12	1 (referent)	1 (referent)	18	1 (referent)	1 (referent)
High	74	23	1.40 (0.70–2.81)	1.71 (0.75–3.91)	29	1.22 (0.68–2.20)	1.33 (0.66–2.66)
p			0.35	0.20		0.51	0.43
TB-distance (TB grade 3)	106	44			55		
Low	11	4	1 (referent)	1 (referent)	5	1 (referent)	1 (referent)
High	95	40	1.39 (0.50–3.90)	1.29 (0.38–4.33)	50	1.46 (0.58–3.68)	1.41 (0.50–3.97)
p			0.53	0.68		0.42	0.51
Cohort 2							
TB-distance (all tumors)	1,063	296			531		
Low	873	204	1 (referent)	1 (referent)	404	1 (referent)	1 (referent)
High	190	92	2.45 (1.91–3.14)	1.34 (1.04–1.74)	127	1.81 (1.48–2.21)	1.22 (0.99–1.50)
p			< 0.0001	0.026		< 0.0001	0.067
TB-distance (TB grade 1)	803	175			359		
Low	773	164	1 (referent)	1 (referent)	342	1 (referent)	1 (referent)
High	30	11	1.86 (1.01–3.42)	1.61 (0.86–3.04)	17	1.46 (0.89–2.37)	1.20 (0.73–1.97)
p			0.047	0.14		0.13	0.48
TB-distance (TB grade 2)	148	61			94		
Low	77	30	1 (referent)	1 (referent)	49	1 (referent)	1 (referent)
High	71	31	1.05 (0.63–1.73)	0.93 (0.54–1.62)	45	0.93 (0.62–1.40)	0.91 (0.59–1.41)
p			0.86	0.81		0.74	0.66
TB-distance (TB grade 3)	112	60			78		
Low	23	10	1 (referent)	1 (referent)	13	1 (referent)	1 (referent)
High	89	50	1.34 (0.68–2.65)	1.30 (0.61–2.77)	65	1.37 (0.75–2.49)	1.26 (0.65–2.46)
p			0.39	0.50		0.30	0.49

Abbreviations: CI, confidence interval; HR, hazard ratio; TB, tumor budding

Multivariable Cox proportional hazards regression models were adjusted for sex, age (< 65, 65–75, > 75), year of operation (Cohort 1: 2006–2010, 2011–2015, 2016–2020; Cohort 2: 2000–2005, 2006–2010, 2011–2015), tumor location (proximal colon, distal colon, rectum), disease stage (I–II, III, IV), WHO grade (Low-grade, High-grade), lymphovascular invasion (no, yes), mismatch repair (MMR) status (proficient, deficient), and *BRAF* status (wild-type, mutant)

To assess whether TB-distance adds prognostic information beyond conventional tumor budding, we evaluated TB-distance within ITBCC tumor budding grade strata (Table 2). Within TB-grade subgroups, associations were not statistically significant in most analyses. A nominal association with shorter CSS was observed in the tumor budding grade 1 subgroup in univariable analyses (HR 1.86 95% CI 1.01–3.42, p = 0.047) (Table 2, Tables S2-S4). In a direct comparison including both variables in the same Cox model, conventional tumor budding remained associated with outcome, whereas TB-distance did not provide additional prognostic information beyond tumor budding (Table S5).

## Discussion

We evaluated a novel tumor budding metric in CRC, defined as the distance that farthest tumor buds extend away from the tumor bulk (TB-distance). High TB-distance was associated with adverse clinicopathologic features and shorter survival in two independent cohorts. However, when conventional tumor budding grades were accounted for, TB-distance did not provide additional prognostic information. These findings suggest that TB-distance largely reflects the same invasive phenotype captured by established ITBCC tumor budding assessment.

High TB-distance was associated with features of aggressive disease such as advanced stage, lymphovascular invasion, and high conventional tumor budding. The particularly strong association with tumor budding supports the interpretation that TB-distance is closely coupled with overall degree of tumor cell dissociation at the invasive front. Overall, associations with adverse prognostic features seen for TB-distance mirror associations consistently reported for conventional tumor budding studies [15, 19–21].

To our knowledge, spatial dispersion of tumor buds away from the main tumor bulk has not been previously studied as a standalone variable. A biologically plausible hypothesis was that buds found farther from the main tumor mass could represent more advanced invasive behavior, potentially linked to partial epithelial-to-mesenchymal transition and related invasion programs [22]. Recent work supports conceptual links between tumor budding, poorly differentiated clusters, and partial epithelial-to-mesenchymal transition, with tumor budding reflecting a more advanced stage of CRC invasion [23]. While we hypothesized that tumor buds extending deeper into the invasive margin might exhibit invasion-related biological features to a greater extent than buds that remain closer to the tumor bulk, this hypothesis was not supported by prognostic data, as TB-distance did not retain independent prognostic value when conventional tumor budding was included in the same model. This negative result is informative because it reinforces that clinically relevant prognostic information is primarily conveyed by conventional budding assessment rather than by spatial dispersion alone.

Although TB-distance did not add prognostic value beyond conventional tumor budding, it may still be useful in practice. Widely dispersed buds can function as a simple visual prompt to re-scan the invasive front for a higher-density budding hotspot, supporting consistent application of ITBCC scoring. In addition, TB-distance represents a simple spatial feature that may be well suited for computational pathology workflows. Automated methods for tumor budding assessment and related invasive margin features are advancing rapidly [24–28], and spatial dispersion metrics could complement automated bud detection by providing quantitative descriptors of invasive growth patterns.

This study has limitations. The assessment of tumor budding as well as TB-distance is observer dependent, and reproducibility for TB-distance was moderate. However, this level of agreement is comparable to prior reports on interobserver reproducibility of conventional tumor budding, assessed using ITBCC criteria [29]. Exclusion of patients who received neoadjuvant therapy may limit generalizability, particularly to rectal cancer and other pretreated tumors. Finally, the strong correlation between TB-distance and conventional tumor budding implies collinearity, which likely contributes to the limited incremental prognostic value observed in joint models. Strengths of this study include the large, multicohort design, comprising more than 1,800 cases, and comprehensive clinicopathologic annotation, which enabled reliable multivariable survival analyses.

In conclusion, the distance that tumor buds extend from the tumor bulk correlates with aggressive clinicopathologic features and adverse outcomes, but it does not independently impact CRC prognosis beyond conventional tumor budding assessment. TB-distance may nonetheless serve as a useful visual aid for pathologists when assessing tumor budding and a candidate spatial feature for future computational pathology approaches to invasive front phenotyping.

## Supplementary Information

Below is the link to the electronic supplementary material.

ESM 1 (PDF 320 KB)

## Data Availability

Data generated and/or analyzed during this study are not publicly available. The sharing of data will require approval from relevant ethics committees and/or biobanks. Further information including the procedures to obtain and access data of Finnish Biobanks are described at https://finbb.fi/en/fingenious-service.

## Abbreviations

CRC: Colorectal cancer
MMR: Mismatch-repair
ITBCC: The International Tumor Budding Consensus Conference
CSS: Cancer-specific survival
OS: Overall survival
ROC: Receiver-operating characteristic
AUC: Area under the curve
