# Supplementary material for: Distance-based evaluation of tumor budding in colorectal cancer
Source: Virchows Arch. 2026 Mar 6;489(4):887–95. doi: 10.1007/s00428-026-04471-9 (PMC13714054; doi:10.1007/s00428-026-04471-9)
Supplement: Supplementary file 1 [file 428_2026_4471_MOESM1_ESM.pdf]

Supplementary tables

Table S1. Multivariable Cox proportional hazards regression models for cancer-specific survival and overall survival according to TB-distance in Cohorts 1 and 2.

	Cohort 1		Cohort 2	
	Cancer-specific survival	Overall survival	Cancer-specific survival	Overall survival
	Multivariable HR (95% CI)	Multivariable HR (95% CI)	Multivariable HR (95% CI)	Multivariable HR (95% CI)
TB-distance				
Low	1 (referent)	1 (referent)	1 (referent)	1 (referent)
High	1.47 (1.04-2.09)	1.37 (1.04-1.81)	1.34 (1.04-1.74)	1.22 (0.99-1.50)
Age				
<65	1 (referent)	1 (referent)	1 (referent)	1 (referent)
65–75	1.65 (1.09-2.50)	1.72 (1.23-2.47)	1.14 (0.85-1.53)	1.32 (1.03-1.69)
>75	2.63 (1.72-4.02)	4.35 (3.12-6.06)	1.79 (1.34-2.39)	2.92 (2.32-3.68)
Sex				
Male	1 (referent)	1 (referent)	1 (referent)	1 (referent)
Female	1.00 (0.72-1.39)	0.84 (0.66-1.06)	0.86 (0.68-1.09)	0.75 (0.63-0.90)
Year of operation				
2000–2005	–	–	1 (referent)	1 (referent)
2006–2010	1 (referent)	1 (referent)	0.62 (0.47-0.82)	0.70 (0.57-0.87)
2011–2015	0.91 (0.61-1.36)	0.89 (0.66-1.20)	0.50 (0.37-0.66)	0.63 (0.51-0.78)
2016–2020	0.58 (0.38-0.88)	0.67 (0.49-0.92)	–	–
Tumor location				
Proximal colon	1 (referent)	1 (referent)	1 (referent)	1 (referent)
Distal colon	1.21 (0.80-1.83)	1.11 (0.81-1.52)	0.89 (0.68-1.16)	0.95 (0.78-1.16)
Rectum	1.02 (0.67-1.55)	1.04 (0.76-1.41)	0.81 (0.56-1.17)	0.87 (0.66-1.15)
UICC disease stage				
I–II	1 (referent)	1 (referent)	1 (referent)	1 (referent)
III	2.72 (1.65-4.48)	1.22 (0.90-1.65)	3.12 (2.27-4.28)	1.50 (1.22-1.85)
IV	19.1 (11.3-32.4)	7.65 (5.34-11.0)	17.9 (12.8-25.1)	7.88 (6.17-10.1)
WHO grade				
Low-grade	1 (referent)	1 (referent)	1 (referent)	1 (referent)
High-grade	1.32 (0.86-2.03)	1.08 (0.76-1.53)	1.60 (1.20-2.13)	1.69 (1.36-2.11)
Lymphovascular invasion				
No	1 (referent)	1 (referent)	1 (referent)	1 (referent)
Yes	1.94 (1.26-2.99)	1.33 (1.00-1.76)	1.79 (1.39-2.30)	1.51 (1.23-1.86)
MMR status				
MMR proficient	1 (referent)	1 (referent)	1 (referent)	1 (referent)
MMR deficient	0.59 (0.28-1.23)	1.02 (0.66-1.56)	0.59 (0.35-0.97)	0.69 (0.49-0.98)
BRAF mutation				
Wild-type	1 (referent)	1 (referent)	1 (referent)	1 (referent)
Mutant	1.37 (0.76-2.45)	1.23 (0.81-1.85)	1.38 (0.90-2.11)	1.49 (1.09-2.03)

Abbreviations: CI, confidence interval; UICC, Union for International Cancer Control; MMR, mismatch repair

Table S2. Multivariable Cox proportional hazards regression models for cancer-specific survival and overall survival according to TB-distance in Cohorts 1 and 2 for tumors with low ITBCC tumor budding grade (0-4 buds).

	Cohort 1		Cohort 2	
	Cancer-specific survival	Overall survival	Cancer-specific survival	Overall survival
	Multivariable HR (95% CI)	Multivariable HR (95% CI)	Multivariable HR (95% CI)	Multivariable HR (95% CI)
TB-distance				
Low	1 (referent)	1 (referent)	1 (referent)	1 (referent)
High	0.99 (0.41-2.39)	0.97 (0.54-1.74)	1.61 (0.86-3.04)	1.20 (0.73-1.97)
Age				
<65	1 (referent)	1 (referent)	1 (referent)	1 (referent)
65–75	1.50 (0.79-2.84)	1.83 (1.13-2.96)	1.35 (0.92-2.00)	1.52 (1.11-2.08)
>75	3.55 (1.84-6.88)	5.20 (3.31-8.17)	1.93 (1.30-2.85)	3.38 (2.52-4.54)
Sex				
Male	1 (referent)	1 (referent)	1 (referent)	1 (referent)
Female	0.79 (0.49-1.27)	0.73 (0.54-0.99)	0.84 (0.61-1.15)	0.65 (0.52-0.81)
Year of operation				
2000–2005	–	–	1 (referent)	1 (referent)
2006–2010	1 (referent)	1 (referent)	0.68 (0.47-0.99)	0.78 (0.61-1.01)
2011–2015	0.92 (0.48-1.75)	0.92 (0.62-1.36)	0.48 (0.33-0.70)	0.67 (0.52-0.88)
2016–2020	0.62 (0.32-1.21)	0.77 (0.51-1.16)	–	–
Tumor location				
Proximal colon	1 (referent)	1 (referent)	1 (referent)	1 (referent)
Distal colon	1.86 (0.96-3.61)	1.22 (0.81-1.84)	0.93 (0.66-1.32)	1.02 (0.80-1.31)
Rectum	1.82 (0.95-3.48)	1.24 (0.83-1.86)	0.87 (0.53-1.42)	0.87 (0.61-1.22)
UICC disease stage				
I–II	1 (referent)	1 (referent)	1 (referent)	1 (referent)
III	3.02 (1.62-5.62)	1.20 (0.82-1.75)	3.36 (2.25-5.02)	1.43 (1.11-1.85)
IV	21.4 (10.6-43.3)	6.80 (4.17-11.1)	25.1 (16.5-38.1)	9.35 (6.94-12.6)
WHO grade				
Low-grade	1 (referent)	1 (referent)	1 (referent)	1 (referent)
High-grade	2.15 (1.09-4.24)	1.24 (0.75-2.05)	2.43 (1.62-3.64)	1.96 (1.46-2.65)
Lymphovascular invasion				
No	1 (referent)	1 (referent)	1 (referent)	1 (referent)
Yes	1.36 (0.77-2.39)	1.13 (0.79-1.61)	1.76 (1.25-2.46)	1.46 (1.11-1.91)
MMR status				
MMR proficient	1 (referent)	1 (referent)	1 (referent)	1 (referent)
MMR deficient	0.78 (0.30-2.03)	1.26 (0.76-2.08)	0.65 (0.35-1.20)	0.78 (0.52-1.17)
BRAF mutation				
Wild-type	1 (referent)	1 (referent)	1 (referent)	1 (referent)
Mutant	0.65 (0.23-1.82)	0.83 (0.49-1.40)	0.84 (0.46-1.51)	1.19 (0.80-1.76)

Abbreviations: CI, confidence interval; UICC, Union for International Cancer Control; MMR, mismatch repair

Table S3. Multivariable Cox proportional hazards regression models for cancer-specific survival and overall survival according to TB-distance in Cohorts 1 and 2 for tumors with intermediate ITBCC tumor budding grade (5-9 buds).

	Cohort 1		Cohort 2	
	Cancer-specific survival	Overall survival	Cancer-specific survival	Overall survival
	Multivariable HR (95% CI)	Multivariable HR (95% CI)	Multivariable HR (95% CI)	Multivariable HR (95% CI)
TB-distance				
Low	1 (referent)	1 (referent)	1 (referent)	1 (referent)
High	1.71 (0.75-3.91)	1.33 (0.66-2.66)	0.93 (0.54-1.62)	0.91 (0.59-1.41)
Age				
<65	1 (referent)	1 (referent)	1 (referent)	1 (referent)
65–75	2.30 (0.92-5.76)	2.21 (0.97-5.06)	1.21 (0.58-2.51)	1.23 (0.67-2.26)
>75	3.04 (1.08-8.53)	5.98 (2.54-14.1)	2.40 (1.20-4.82)	2.89 (1.64-5.09)
Sex				
Male	1 (referent)	1 (referent)	1 (referent)	1 (referent)
Female	0.73 (0.31-1.70)	0.89 (0.45-1.76)	0.94 (0.52-1.69)	1.03 (0.64-1.66)
Year of operation				
2000–2005	–	–	1 (referent)	1 (referent)
2006–2010	1 (referent)	1 (referent)	0.32 (0.15-0.71)	0.37 (0.20-0.67)
2011–2015	0.89 (0.31-2.62)	1.43 (0.59-3.48)	0.66 (0.36-1.21)	0.67 (0.40-1.11)
2016–2020	0.40 (0.15-1.08)	0.55 (0.23-1.30)	–	–
Tumor location				
Proximal colon	1 (referent)	1 (referent)	1 (referent)	1 (referent)
Distal colon	1.70 (0.70-4.15)	1.69 (0.80-3.57)	1.19 (0.65-2.18)	1.14 (0.71-1.82)
Rectum	0.69 (0.22-2.20)	0.95 (0.38-2.36)	0.76 (0.34-1.71)	0.80 (0.41-1.55)
UICC disease stage				
I–II	1 (referent)	1 (referent)	1 (referent)	1 (referent)
III	2.71 (0.74-9.88)	1.67 (0.72-3.85)	2.10 (1.01-4.38)	1.44 (0.83-2.48)
IV	29.4 (7.43-117)	15.0 (5.75-39.4)	12.6 (5.45-29.3)	7.44 (3.81-14.5)
WHO grade				
Low-grade	1 (referent)	1 (referent)	1 (referent)	1 (referent)
High-grade	0.75 (0.27-2.09)	0.89 (0.38-2.08)	1.27 (0.63-2.57)	1.46 (0.81-2.60)
Lymphovascular invasion				
No	1 (referent)	1 (referent)	1 (referent)	1 (referent)
Yes	8.92 (1.12-70.9)	2.85 (1.06-7.61)	1.43 (0.81-2.51)	1.20 (0.74-1.92)
MMR status				
MMR proficient	1 (referent)	1 (referent)	1 (referent)	1 (referent)
MMR deficient	1.48 (0.32-6.90)	0.83 (0.19-3.55)	1.07 (0.30-3.87)	1.06 (0.42-2.67)
BRAF mutation				
Wild-type	1 (referent)	1 (referent)	1 (referent)	1 (referent)
Mutant	1.60 (0.47-5.42)	1.44 (0.51-4.07)	1.13 (0.43-2.97)	1.19 (0.59-2.40)

Abbreviations: CI, confidence interval; UICC, Union for International Cancer Control; MMR, mismatch repair

Table S4. Multivariable Cox proportional hazards regression models for cancer-specific survival and overall survival according to TB-distance in Cohorts 1 and 2 for tumors with high ITBCC tumor budding grade (≥10 buds) .

	Cohort 1		Cohort 2	
	Cancer-specific survival	Overall survival	Cancer-specific survival	Overall survival
	Multivariable HR (95% CI)	Multivariable HR (95% CI)	Multivariable HR (95% CI)	Multivariable HR (95% CI)
TB-distance				
Low	1 (referent)	1 (referent)	1 (referent)	1 (referent)
High	1.29 (0.38-4.33)	1.41 (0.50-3.97)	1.30 (0.61-2.77)	1.26 (0.65-2.46)
Age				
<65	1 (referent)	1 (referent)	1 (referent)	1 (referent)
65–75	1.76 (0.79-3.89)	1.91 (0.92-3.97)	0.74 (0.37-1.48)	0.84 (0.43-1.62)
>75	3.74 (1.58-8.86)		1.52 (0.78-2.96)	2.18 (1.20-3.99)
Sex				
Male	1 (referent)	1 (referent)	1 (referent)	1 (referent)
Female	1.70 (0.88-3.27)	1.26 (0.71-2.23)	1.17 (0.63-1.96)	1.17 (0.71-1.93
Year of operation				
2000–2005	–	–	1 (referent)	1 (referent)
2006–2010	1 (referent)	1 (referent)	0.60 (0.32-1.16)	0.66 (0.38-1.15)
2011–2015	0.77 (0.33-1.80)	0.60 (0.28-1.30)	0.45 (0.21-0.96)	0.54 (0.27-1.07)
2016–2020	0.56 (0.24-1.32)	0.56 (0.27-1.16)	–	–
Tumor location				
Proximal colon	1 (referent)	1 (referent)	1 (referent)	1 (referent)
Distal colon	0.38 (0.14-0.99)	0.42 (0.18-0.99)	0.80 (0.44-1.48)	0.69 (0.40-1.20)
Rectum	0.59 (0.27-1.29)	0.78 (0.39-1.54)	0.59 (0.23-1.50)	0.78 (0.37-1.65)
UICC disease stage				
I–II	1 (referent)	1 (referent)	1 (referent)	1 (referent)
III	2.72 (0.82-9.06)	1.59 (0.64-4.00)	1.89 (1.09-7.66)	2.23 (1.04-4.81)
IV	16.4 (4.99-54.0)	10.3 (4.08-26.2)	10.1 (3.73-27.4)	8.21 (3.64-18.5)
WHO grade				
Low-grade	1 (referent)	1 (referent)	1 (referent)	1 (referent)
High-grade	1.09 (0.52-2.30)	1.05 (0.53-2.06)	0.87 (0.46-1.66)	1.30 (0.75-2.25)
Lymphovascular invasion				
No	1 (referent)	1 (referent)	1 (referent)	1 (referent)
Yes	1.78 (0.63-5.01)	1.26 (0.59-2.70)	1.86 (1.05-3.29)	1.86 (1.12-3.11)
MMR status				
MMR proficient	1 (referent)	1 (referent)	1 (referent)	1 (referent)
MMR deficient	*	0.22 (0.034-1.36)	0.53 (0.10-2.90)	0.56 (0.14-2.21)
BRAF mutation				
Wild-type	1 (referent)	1 (referent)	1 (referent)	1 (referent)
Mutant	9.47 (3.00-29.8)	9.50 (3.28-27.5)	6.51 (2.64-16.0)	5.29 (2.31-12.1)

Abbreviations: CI, confidence interval; UICC, Union for International Cancer Control; MMR, mismatch repair. *Insufficient data for analysis, no mortality endpoints in this category

Table S5. Comparison of the prognostic power of TB-distance and tumor budding using Cox regression models for cancer-specific survival.

Variable	No. of cases	No. of events	Model 1 (Univariable) HR (95% CI)	Model 2 (multivariable) HR (95% CI)	Model 3 (multivariable) HR (95% CI)
**Cohort 1**					
TB-distance	771	155			
Low (<123 µm)	565	85	1 (referent)	1 (referent)	1 (referent)
High (≥123 µm)	206	70	2.59 (1.89-3.56)	1.41 (0.89-2.23)	1.04 (0.65-1.65)
P			<0.0001	0.15	0.87
ITBCC tumor budding	771	155			
Grade 1 (0-4)	538	76	1 (referent)	1 (referent)	1 (referent)
Grade 2 (5-9)	127	35	2.13 (1.43-3.18)	1.78 (1.11-2.86)	1.56 (0.98-2.48)
Grade 3 (≥10)	106	44	3.68 (2.54-5.34)	2.80 (1.65-4.73)	1.83 (1.07-3.13)
P_{trend}			<0.0001	0.00013	0.024
**Cohort 2**					
TB-distance	1,063	296			
Low (<123 µm)	873	204	1 (referent)	1 (referent)	1 (referent)
High (≥123 µm)	190	92	2.45 (1.91-3.14)	1.31 (0.92-1.87)	1.20 (0.85-1.71)
P			<0.0001	0.13	0.30
ITBCC tumor budding	1,063	296			
Grade 1 (0-4)	803	175	1 (referent)	1 (referent)	1 (referent)
Grade 2 (5-9)	148	61	2.24 (1.68-3.00)	1.96 (1.39-2.77)	1.22 (0.86-1.73)
Grade 3 (≥10)	112	60	3.20 (2.39-4.30)	2.60 (1.73-3.89)	1.17 (0.77-1.78)
P_{trend}			<0.0001	<0.0001	0.42

Model 2: Cox proportional hazards regression model that included TB-distance and tumor budding.

Model 3: Cox proportional hazards regression model that included TB-distance and tumor budding and was additionally adjusted for age (<65, 65-75, >75), sex (male, female), stage (I-II, III, IV), tumor location (proximal colon, distal colon, rectum), year of operation (Cohort 1: 2006–2010, 2011–2015, 2016–2020; Cohort 2: 2000–2005, 2006–2010, 2011–2015), lymphovascular invasion (no, yes), grade (low-grade, high-grade), MMR status (proficient, deficient) and BRAF (wild-type, mutant). Abbreviations: HR, hazard ratio; CI, confidence interval

Supplementary figures

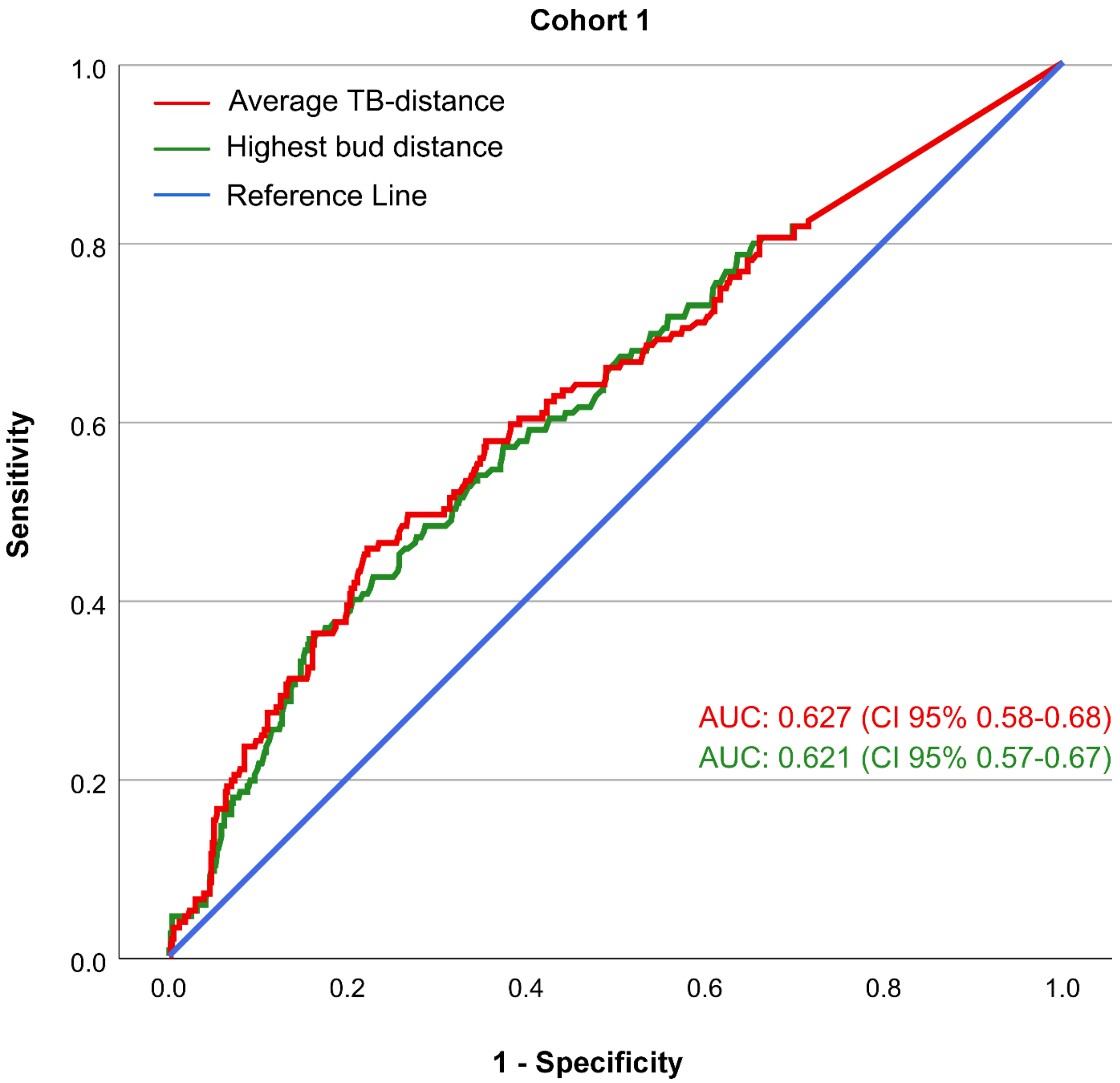

Figure S1. Receiver-operating characteristic (ROC) analysis of TB-distance in predicting cancer-specific mortality in Cohort 1. The red curve shows TB-distance defined as the mean distance from the tumor bulk to the three farthest tumor buds. The green curve shows the distance measured using only the single farthest tumor bud. Analyses include Cohort 1 patients after excluding neoadjuvant treated cases. For ROC derivation, 10-year CSS status was coded as cancer-related death within 10 years versus no cancer-related death, with patients censored before 10 years classified as event-free.
